# Resistome and microbiome profiling of bovine milk following antimicrobial dry cow therapy: insights from short- and long-read metagenomic sequencing

**DOI:** 10.3389/frmbi.2025.1672438

**Published:** 2025-10-10

**Authors:** Leire Urrutia-Angulo, José Luis Lavín, Beatriz Oporto, Gorka Aduriz, Ana Hurtado, Medelin Ocejo

**Affiliations:** ^1^ Animal Health Department, NEIKER − Basque Institute for Agricultural Research and Development, Basque Research and Technology Alliance (BRTA), Derio, Bizkaia, Spain; ^2^ Applied Mathematics Department, NEIKER − Basque Institute for Agricultural Research and Development, Basque Research and Technology Alliance (BRTA), Derio, Bizkaia, Spain

**Keywords:** mastitis, dairy cattle, dry cow therapy, antimicrobial resistance, milk resistome, shotgun metagenomics, short-read sequencing, long-read sequencing

## Abstract

Selective antimicrobial dry cow therapy (DCT) is implemented as part of mastitis control programs, particularly in dairy cows with recent clinical episodes or elevated somatic cell counts. In this study, we investigated the effects of the use of antimicrobials at drying-off on the milk microbiota and resistome by comparing treated (T, n=18) and untreated (NT, n=13) cows. Milk samples from all animals were analyzed using short-read Illumina shotgun sequencing and a subset of 10 samples were also subjected to long-read Oxford Nanopore Technologies (ONT) sequencing. No significant differences in microbial composition or diversity were observed between treated and untreated groups with either technique, indicating that antimicrobial DCT may not induce long-term shifts in the milk microbiota. However, cows receiving antibiotic treatment showed a higher diversity and abundance of genetic determinants of resistance (GDRs) in their milk resistome. Findings from the two sequencing platforms revealed limited concordance in antimicrobial resistance gene content, highlighting that sequencing platform and bioinformatic pipeline choices substantially influence resistome profiling outcomes. Furthermore, the high proportion of host DNA limited sequencing depth and sensitivity, underscoring the need for improved host DNA depletion or targeted enrichment strategies. This study provides insights into the biological and methodological challenges of milk resistome characterization, particularly in low-biomass, host-DNA-rich samples and demonstrates the lack of standardized analytical approaches in resistome studies. Overall, our findings support the prudent use of antibiotics and highlight the need for further longitudinal studies to clarify the temporal dynamics of antimicrobial DCT effects on the milk resistome and microbiota.

## 1 Introduction

Mastitis is one of the most prevalent and economically significant diseases in dairy farming, adversely affecting both milk production and animal welfare. The dry period, a non-lactating interval typically lasting around 60 days before calving, is particularly vulnerable to new intramammary infections (Bradley and Green, 2004) but also represents a strategic window to clear existing infections and to allow the udder to recover for the next lactation cycle (Bradley and Green, 2004). To mitigate mastitis risk during this period, dry cow therapy (DCT) traditionally involved routine prophylactic antibiotic administration to all cows at drying-off. Although this practice has been effective in reducing new infections, it has also raised major concerns regarding the emergence and spread of antimicrobial resistance (AMR), particularly since the antibiotics commonly used in DCT (often involving β-lactams and macrolides), are also critical for human medicine (World Health Organization, 2024). In response, the European Union (Regulation UE 2019/6) has restricted antimicrobial use in livestock, banning prophylactic treatment unless the risk of infection is very high and the outcomes potentially severe. This has shifted industry practice towards selective DCT, where antibiotics are only administered to cows with mastitis or at high risk of developing the disease.

The impact of antimicrobial DCT on the milk microbiota is still not fully understood. Some studies have reported increased microbial diversity and changes in taxonomic profiles and antimicrobial resistance genes (ARG) abundance following DCT (Patangia et al., 2023; Vasco et al., 2023), while others found no significant differences in overall composition or diversity indices between treated and untreated cows (Biscarini et al., 2020; Bonsaglia et al., 2017; Pollock et al., 2021; Vasquez et al., 2022). These inconsistencies could be attributed to the timing of sampling, the specificity of the antimicrobials used (which may eliminate pathogens while sparing commensals) or to the inherent stability and resilience of the mammary gland microbiome (Biscarini et al., 2020; Ganda et al., 2016). Such discrepancies may also arise from differences in the experimental design and the methodology used for microbiota characterization such as sequencing technology, sequencing depth or bioinformatic tools employed for the analysis. Beyond taxonomic profiling, special attention should also be given to the milk resistome, which encompasses the full collection of genetic determinants of resistance (GDRs), such as resistance genes and single nucleotide point mutations (SNPs) that confer resistance to antimicrobials, metals, and disinfectants. Antimicrobial use during DCT can also exert selective pressure on the milk microbiota, potentially promoting the emergence of resistant populations and maintenance of ARGs, which may ultimately enter the food chain. Therefore, milk resistome studies (Perry et al., 2014) are essential to understand how DCT influences the dynamics of antimicrobial resistance in milk.

Shotgun metagenomic sequencing enables a comprehensive taxonomic and functional profiling of the microbiota by sequencing all the DNA present in a sample, including ARGs. In this study, we applied shotgun metagenomic sequencing to investigate the impact of antimicrobial DCT on the microbial taxonomic composition and resistome of bovine milk using Illumina short-read sequencing. Additionally, we sequenced a subset of samples using Oxford Nanopore Technologies (ONT) and examined how its results aligned with those from Illumina short-read sequencing using a unified bioinformatic pipeline. By integrating taxonomic and resistome profiling across two widely used sequencing platforms, we aim to better understand how antibiotic use during the dry period influences the microbial ecosystem of dairy cattle milk, gain insight about biological and methodological challenges and to explore how sequencing and bioinformatic analysis choices can shape resistome profiling outcomes of complex, low-biomass samples such as milk.

## 2 Materials and methods

### 2.1 Study farm and sample collection

The study was carried out in a single Holstein-Friesian cattle farm, with around 650 animals in lactation, located in Navarra (Spain). Animals were housed in open lots with a roofed area. The bedding in the covered free-stall area is made up of straw, which is tilled with a rototiller to aerate the top layer (15-25cm). The open recreational zone has a concrete floor where manure accumulates, which is removed periodically.

Lactating cows are milked twice a day and dried off approximately 94 days before the expected calving date. At drying-off, a non-antibiotic teat sealant is applied to all the animals. Intramammary antibiotic ointment was selectively applied only to cows with a history of clinical mastitis or with somatic cell counts (SCC) above 200,000 cells/mL in any of the last five monthly controls. The product was applied on the last day of milking, prior to drying.

Milk samples were collected in November 2023 from 31 multiparous, clinically healthy cows. Cows were categorized as either treated with antibiotics (T; n = 18) or non-treated (NT; n=13) at drying-off. In this study, cows receiving antimicrobial DCT were treated with one of the following intramammary products: Virbactan 150mg (Cefquinome 150 mg - Virbac, n=13), Cefquitan 75 mg (Cefquinome 75mg - Fatro, n=1), or Mamyzin Secado (Penetamate iohydrate 100 mg, Banzylpenicillin benetamine 280 mg, and Framycetin sulfate 100 mg - Boehringer Ingelheim, n=4). NT cows had not received any intramammary antimicrobial treatment during the ongoing lactation or the immediately preceding one. Each animal was sampled once. NT animals were sampled at an average of 109 days in lactation (DIL), and T animals at an average of 159 DIL (**Supplementary Table S1**), approximately 260 days after the antibiotic treatment for the T group. From each animal, a composite sample (30 mL) was manually collected by aseptically drawing approximately equal volumes from all four udder quarters, following a protocol based on National Mastitis Council (NMC) guidelines (teat disinfection, discarding of foremilk, and aseptic collection). The samples were then homogenized, refrigerated at 4°C, and processed within 24 hours of collection.

### 2.2 DNA extraction and shotgun sequencing

Upon arrival at the laboratory, samples were first subjected to a skimming pretreatment involving centrifugation at 4,500 × g for 30 min at 4°C to remove the supernatant containing the fat layer. The resulting pellet was washed with sterile phosphate-buffered saline (1× PBS) and centrifuged again at 10,000 × g for 10 min at 4°C. The final pellet was then resuspended in DNA/RNA Shield (Zymo Research Corp.). The DNA of all samples was extracted using the ZymoBIOMICS DNA 96 MagBead Kit (Zymo Research Corp.) on the Kingfisher Flex robot (Thermo Scientific), following the manufacturer’s instructions. Extracted DNA was concentrated using a SpeedVac (Thermo Scientific) to reach the required concentration for sequencing (≥10ng/µL). DNA concentration and purity were determined using a NanoDrop 1000 Spectrophotometer (Thermo Scientific) and in a Qubit 2.0 fluorimeter (Invitrogen), using Qubit double-stranded DNA (dsDNA) high-sensitivity (HS) assay Kit. Additionally, DNA integrity was assessed by electrophoresis on a 0.8% agarose gel. Extracted DNA was stored at -80°C until sequencing.

Shotgun metagenomic sequencing of the 31 DNA samples was performed on an external commercial facility on an Illumina NovaSeq X Plus Series platform (Novogene), generating 2 × 150 bp paired-end reads. In addition, 10 of these samples (5 NT and 5 T) were sequenced in-house using the Rapid Barcoding Kit 24 V14 (SQK-RBK114.24) on a MinION Mk1C device with a FLO-MIN114 (R10.4.1) flow cell (Oxford Nanopore Technologies, ONT) (**Supplementary Table S1**). Samples were selected for ONT sequencing based on availability of sufficient DNA yield and concentration to meet the input requirements for library preparation (200 ng in 10 µL). Library preparation and sequencing parameter settings were carried out according to the manufacturer’s instructions.

### 2.3 Bioinformatic analysis

Illumina raw reads were assessed for quality using FastQC (v.0.12.1) (Andrews, 2010). Downstream processing was conducted using the SqueezeMeta pipeline (Tamames and Puente-Sánchez, 2019) with default settings, which include assembly and taxonomic classification. In brief, reads were assembled with MEGAHIT and taxonomic classification was carried out using DIAMOND for fast sequence alignment against the GenBank non-redundant (nr) database. For resistome characterization, Illumina reads were processed using the AMR++ bioinformatics pipeline (v3.0.6) in conjunction with the MEGARes database (v3.0) (Bonin et al., 2023; Lakin et al., 2017). AMR++ is optimized for use with raw data from high throughput sequencing and metagenomic analysis and provides estimations of the abundance of resistance genes. MEGARes was selected over other databases (e.g., CARD) due to its compatibility with the AMR++ workflow, its streamlined and hierarchical annotation structure, and the inclusion of genes associated with resistance to antimicrobials, metals, and biocides. The pipeline was executed under default parameters, with the optional SNPs verification flag (-snp -Y) enabled. Only GDRs with coverage higher than 80% of the reference nucleotide sequence were retained for further analysis.

The ONT-generated sequences were base-called on high accuracy mode (HAC), and barcodes and adapters were trimmed using Dorado (v7.6.7). Read quality was evaluated using both FastQC and NanoPlot (v1.44.1). Reads were mapped against the *Bos taurus* 8 reference genome using Bowtie2 (v2.5.3) on Galaxy (https://usegalaxy.eu/) to remove host DNA. For taxonomic classification, cleaned reads were processed through SqueezeMeta pipeline using the sqm_longreads.pl utility, run with default parameters. The assembly-based taxonomic profiling approach using SqueezeMeta was chosen to increase classification specificity and reduce false positives while ensuring consistent processing across both short- and long-read datasets.

Concordance in ARG detection between Illumina and ONT sequencing platforms was assessed based on the 10 bovine milk samples sequenced with both methods. For this comparison, ARG identification was conducted using the ABRicate pipeline with the MEGARes database (minimum coverage of 80% and identity threshold of 90%), to ensure consistency in the database used in both analyses. The comparison was restricted to ARGs classified under the “Drug” category that did not require SNP confirmation. Contigs (Illumina) and reads (ONT) carrying ARGs were extracted and subjected to nucleotide alignment using NCBI’s BLASTn. The best hit was retained when the sequence alignment met thresholds of ≥60% query coverage and ≥90% identity.

### 2.4 Statistical analysis

The SQMtools package was used to import and integrate SqueezeMeta output in R (v4.3.2) for taxonomic and diversity analysis. Eukaryotic taxa (in both Illumina and ONT datasets) and taxa with fewer than 10 read counts or present in only a single sample (Illumina dataset) were filtered out. Rarefaction curves were constructed with the ranacapa (v0.1.0) package (Kandlikar et al., 2018) to assess sequencing depth.

Microbial community structure was assessed using both alpha and beta diversity metrics. For alpha diversity, species richness and evenness were quantified using the Chao1 and Shannon indices with phyloseq (v1.46.0) package (McMurdie and Holmes, 2013). Differences among treatment groups were statistically tested using the non-parametric Wilcoxon rank-sum test. To account for variations in sequencing depth, data were normalized using total sum scaling (TSS). To visualize alpha diversity metrics across treatment groups, both boxplots and violin plots were generated with ggplot2 (v3.5.1) and ggstatsplot (v0.12.5) (Patil, 2021; Wickham, 2016). Beta diversity was calculated using Bray–Curtis dissimilarity distances with phyloseq, and differences in community composition between T and NT groups were evaluated through permutational multivariate analysis of variance (PERMANOVA). Principal Coordinates Analysis (PCoA) was conducted with phyloseq and ordination plots were generated using ggplot2 and ggrepel (v0.9.5) (Slowikowski, 2016). To assess the homogeneity of multivariate dispersions, Bray–Curtis distances based on relative abundances were analyzed with the betadisper function from the vegan package (v2.6-4) (Oksanen et al., 2022), followed by a permutation test.

Taxonomic composition was examined at all ranks (from phylum to genus) using the phyloseq and microbiome (v1.24.0) packages (Lahti and Shetty, 2017). For each taxonomic rank, the ten most abundant taxa were visualized using ggplot2, with the remaining taxa grouped under the category “Other”. Additionally, genus-level differential abundance analysis was performed using the ANCOM-BC2 method implemented in the ANCOMBC (v2.4.0) package (Lin and Peddada, 2020), with treatment group specified as a fixed effect, and Holm correction applied. Genera with FDR-adjusted *q*-value < 0.05 and an absolute log-fold change (|lfc|) > 1 were considered differentially abundant and were visualized using customized bar plots created with ggplot2. Core genera, defined as those with a minimum prevalence of 80% across samples and relative abundance of at least 1%, were identified using the microbiomeutilities package (v1.00.17) (Shetty and Lahti, 2024).

Resistome count data (AMR++ output) were normalized using the count-per-million (CPM) method. Logistic regression was used to assess the association between treatment group and the presence/absence of GDRs. Zero-inflated negative binomial (ZINB) regression model was used to compare the normalized GDRs abundance between T and NT, accounting for the high frequency of zero values and overdispersion on the Illumina data. Agreement in ARG detection between Illumina and ONT sequencing platforms were evaluated using Cohen’s kappa coefficient based on binary presence/absence of ARGs using psych package (v2.5.3) (Revelle, 2024).

## 3 Results

### 3.1 Sequencing output and taxonomic profiling with Illumina

Illumina shotgun sequencing of 31 samples yielded a total of 799.2 million paired-end reads (median= 24.8 M per sample; IQR = 23.3-27.4 M), with an average quality score of 38.1 per sample (range = 37.5 - 38.6). After removing eukaryotic taxa and filtering out low-abundance taxa, 190,289,730 reads remained (median of 6,122,638 reads/sample).

**Figure 1:**
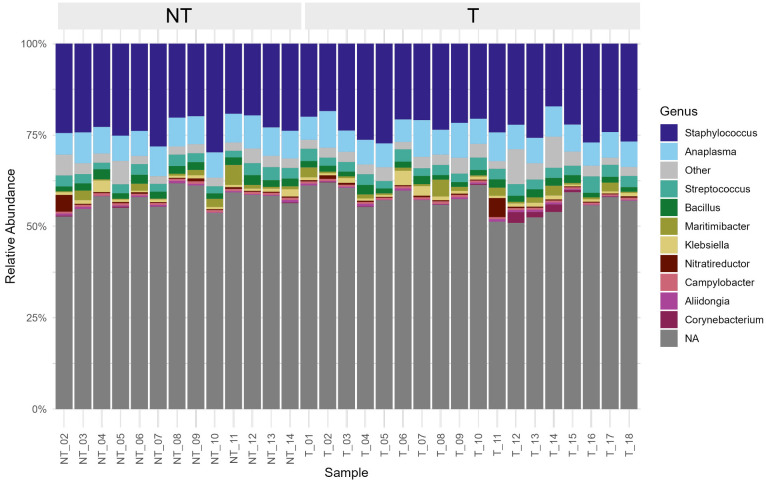
Stacked bar plot showing the relative abundance of bacterial genera in individual milk samples, grouped by dry cow therapy (DCT) treatment. The ten most abundant genera are color-coded as indicated in the legend, while less abundant taxa are grouped under “Other”. Genera that could not be taxonomically classified at genus level are grouped and labelled as “NA”.

Rarefaction curves reached *plateau* at ~2 million reads, indicating sufficient depth to capture most microbial diversity (**Supplementary Figure S1A**). A total of 660 genera were identified, corresponding to 343 families, 169 orders, 78 classes, 39 phyla and 3 kingdoms (Archaea, Bacteria, and Viruses). Unclassified phyla accounted for an average of 50.4% of the sequences per sample and the phyla *Firmicutes, Proteobacteria*, and *Actinobacteria* exhibited relative abundance ≥ 1%, specifically 34.2%, 14.2%, and 1.0%, respectively. Six classified genera were present at an average relative abundance ≥1% (**Supplementary Table S2**; **Figure 1**).

**Figure 2:**
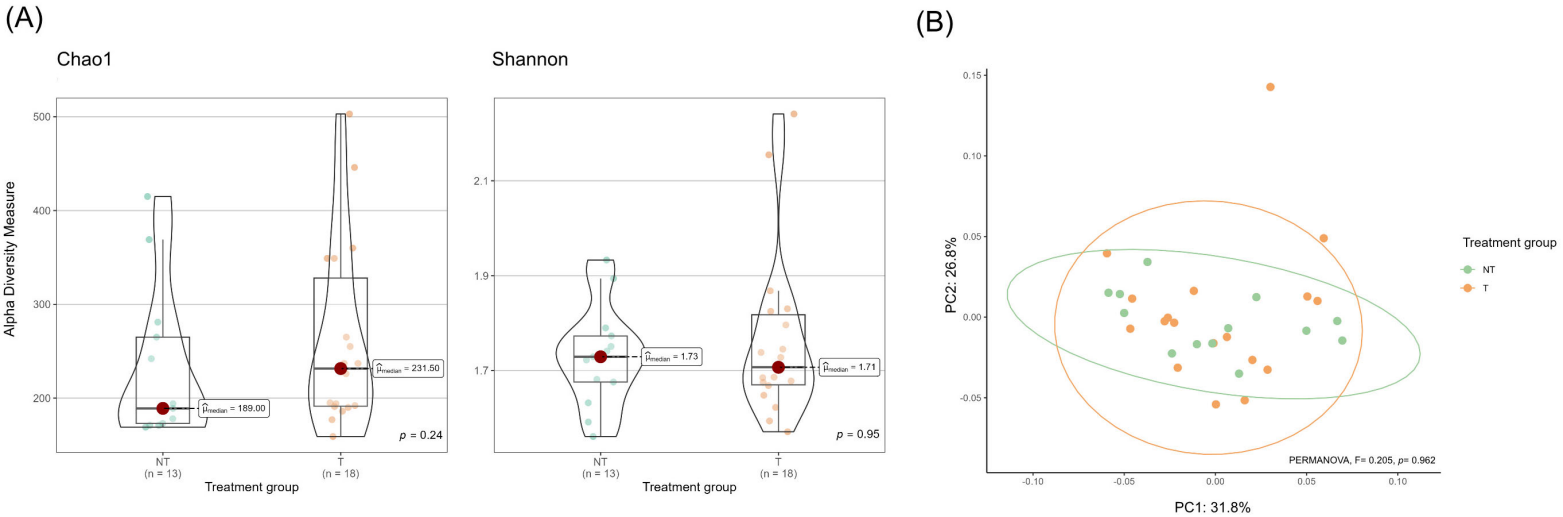
Genus-level milk microbiota diversity in milk collected from dairy cows treated (T) or not treated (NT) with antimicrobials at dry-off. **(A)** Violin plots showing alpha diversity indices at the genus level: richness (Chao1) and evenness (Shannon). Each violin represents the kernel density estimation of alpha diversity measures of individual samples. Overlaid boxplots represent the interquartile range (IQR; Q1 to Q3), with the horizontal line indicating the median. Whiskers extend to values within 1.5×IQR, and dots represent individual samples; values outside this range are plotted as outliers. Statistical comparisons between treatment groups were performed using non-parametric Wilcoxon rank sum test. Pairwise comparisons were adjusted for multiple testing using the false discovery rate (FDR) method. **(B)** Principal Coordinates Analysis (PCoA) based on Bray–Curtis dissimilarity matrix illustrating differences in the genus-level microbial composition of milk samples between T and NT groups. Each point represents an individual sample; colors indicate treatment groups. Ellipses correspond to 95% confidence intervals based on the multivariate t-distribution around the group centroids. Group differences were assessed using PERMANOVA.

Alpha diversity metrics (Chao1 and Shannon indices) did not differ significantly between T and NT animals (**Figure 2A**). Similarly, beta diversity analysis revealed no significant differences in overall community composition between treatment groups (PERMANOVA, F = 0.205, *p* = 0.962; **Figure 2B**). Homogeneity of group dispersions was also similar, with no differences between groups (F = 0.355, *p* = 0.575).

Differential abundance analysis using ANCOM-BC2 identified only one genus (*Turicibacter*) as significantly more abundant in T animals compared to NT ones (lfc > 1, q < 0.05). The core microbiota (≥80% prevalence, ≥1% relative abundance) of both treatment groups was composed by the same four genera, which were present in all samples and corresponded to the most prevalent genera as listed in **Supplementary Table S2**.

### 3.2 Milk resistome profiling with Illumina

**Figure 3:**
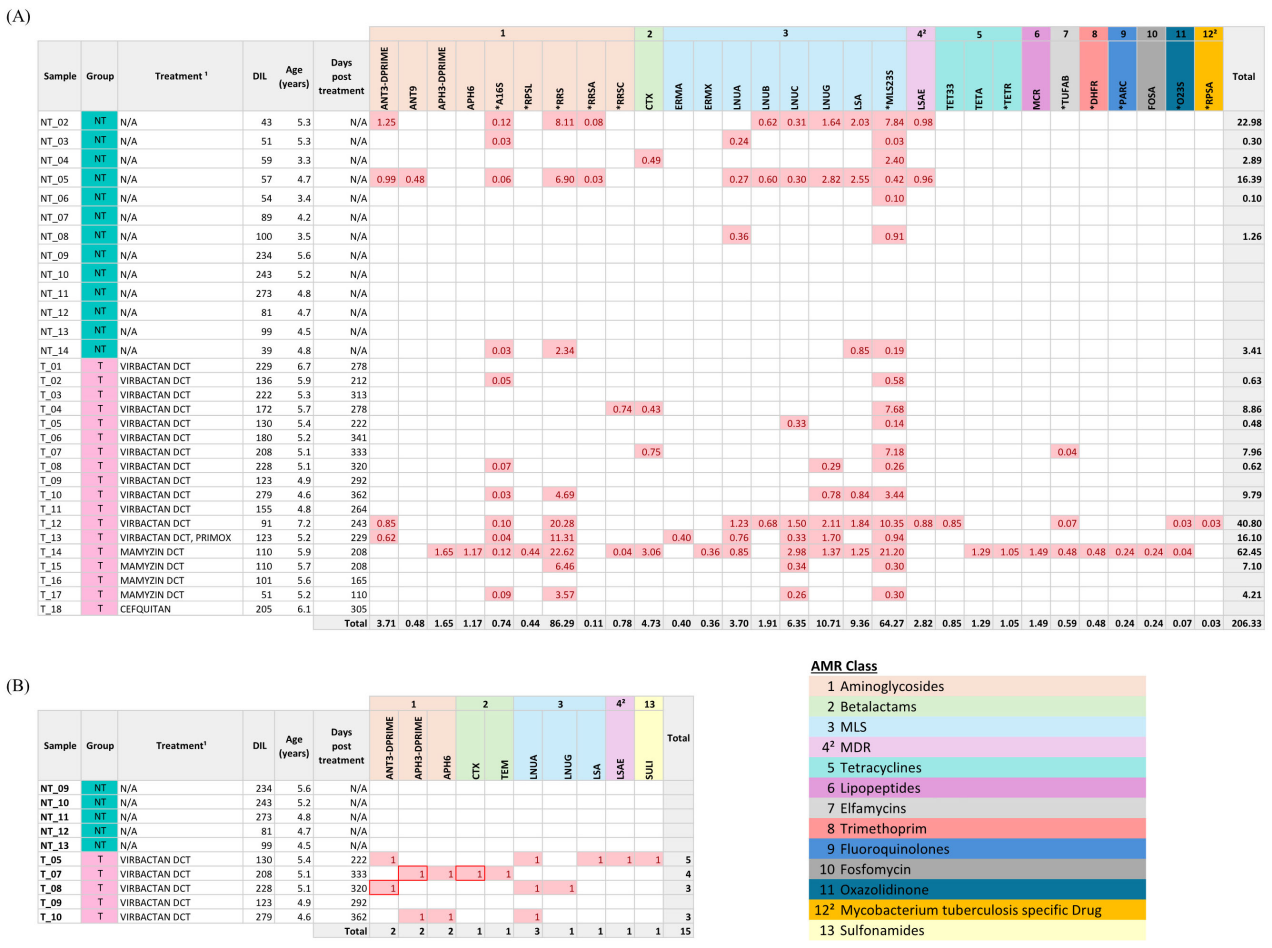
Distribution of genetic determinants of resistance (GDRs) in milk samples from dairy cows. **(A)** Normalized abundance (counts per million, CPM) of GDRs detected by Illumina sequencing using the AMR++ pipeline across milk samples are represented. GDRs are grouped and color-coded by antimicrobial class according to the legend. **(B)** Presence/absence matrix of antimicrobial resistance genes (ARGs) detected in a subset of 10 shared samples using Oxford Nanopore Technologies (ONT) and Illumina sequencing using ABRicate. ARGs identified by both platforms are highlighted with a box. GDRs are grouped and color-coded by antimicrobial class. DIL, Days in lactation; N/A, Not applicable. ¹Active ingredients: Virbactan (cefquinome), Primox (oxytetracycline), Mamyzin (penetamate iodide, benzylpenicillin benethamine, framycetin sulfate), Cefquitan (cefquinome). ²Not classified as antimicrobial classes according to standard criteria. SNP-associated GDRs are marked with an asterisk (*).

A total of 29 GDRs, including 17 ARGs and 12 SNPs, coding for resistance to 10 antimicrobial classes, were detected among the 31 animals (**Figure 3A**). Genetic resistance to macrolide-lincosamide-streptogramin (MLS), aminoglycosides, β-lactams, and tetracyclines were the most prevalent. Seven of the 13 NT animals (53.8%) carried at least one GDR, while 11 of the 18 T animals (61.1%) carried GDRs. Overall, NT animals harbored nine distinct ARGs and four SNPs, associated with resistance to three antimicrobial classes: aminoglycosides, β-lactams, and MLS. T animals carried 16 different ARGs and 11 SNPs, encompassing 10 resistance classes (**Figure 3A**). Eight ARGs and 3 SNPs were common between samples from NT and T animals (**Supplementary Figure S2**). Antimicrobial treatment at drying-off was significantly associated with a higher likelihood of detecting GDRs in milk (logistic regression; OR = 1.61 (1.07 – 2.40), *p* = 0.021) and higher abundance of GDRs compared to untreated cows (ZINB; IRR = 2.43 (1.34 – 4.39), *p* = 0.003).

Genes conferring resistance to heavy metals (n = 18), biocides (n = 2), and both (n = 3) were also identified, corresponding to five different classes of metals (arsenic resistance, copper resistance, mercury resistance, tellurium resistance, and multi-metal resistance) and two classes of biocides (peroxide and multi-biocide resistance). One NT animal carried 1 gene and four T animals carried 1, 2, 6, and 23 genes, respectively (**Supplementary Table S3**). Notably, cow T_14 presented an unusually high number of GDRs (n = 44), including SNPs, AMR, metal, and biocide resistance genes, with 25 of them being unique to this sample.

### 3.3 Sequencing output and taxonomic profiling with ONT sequencing

A subset of ten milk samples was also subjected to metagenomic sequencing using ONT, yielding an average of 1.5 Gb/sample (N50_reads_ = 6,500 bp; average Q = 30.6). Following host DNA removal, non-host DNA accounted for an average of 11.9% of the total reads, which corresponded to a median of 3,522 reads/sample). Prokaryotic sequences were taxonomically assigned to 339 genera, belonging to 145 families, 72 orders, 30 classes, 16 phyla and 3 kingdoms (Archaea, Bacteria, and Viruses), with unclassified phyla accounting for 25.1% of the sequences per sample. Rarefaction curves did not reach *plateau*, indicating that the sequencing depth was insufficient to capture the full microbial diversity present in the samples (**Supplementary Figure S1B**). Alpha diversity (Chao1 and Shannon indices) differed significantly (*p* = 0.02) between T and NT cows, with higher diversity observed in T cows. In contrast, beta diversity did not differ between groups (PERMANOVA, F = 3.30, *p* = 0.067), and no significant differences in dispersion were observed (F = 5.44, *p* = 0.065). Differential abundance analysis at the genus level using ANCOM-BC2 did not identify any significantly different taxa between T and NT animals.

### 3.4 Comparison between Illumina and ONT platforms in ARG detection

For the 10 samples sequenced by both technologies, ARG detection was compared using ABRicate with MEGARes database to enable direct comparison between sequencing platforms. ARGs were exclusively detected in treated animals with both techniques (**Figure 3B**). However, ONT detected a total of 10 ARGs (ANT3-DPRIME, APH3-DPRIME, CTX, APH6, LNUA, LNUG, LSA, LSAE, SULI, TEM) associated to resistance to four antimicrobial classes, whereas Illumina identified only three ARGs (ANT3-DPRIME, APH3-DPRIME, and CTX) encoding resistance for two classes. Of 100 gene-sample combinations (10 samples, 10 ARGs), three showed agreement in ARG presence detection, 85 were concordantly negative by both techniques, and 12 ARG detections were unique to ONT (**Supplementary Table S4**). Overall agreement between platforms in resistome profiling was low (Cohen’s kappa = 0.3; 95% CI: 0.03–0.56).

Microbial host assignment for ARGs-carrying contigs/reads using the defined thresholds (best-matching bacterial species, coverage >60% and identity >96%) was possible for three ONT reads (**Supplementary Table S5**) but not for any of the Illumina contigs.

## 4 Discussion

### 4.1 Milk resistome profiles differed between T and NT cows, whereas no differences in microbiota taxonomic composition were observed

By integrating taxonomic and resistome profiling, this study explored the potential impact of antimicrobial use during the dry period on the milk microbial ecosystem, while also providing complementary insights and methodological considerations associated with the use of different sequencing technologies in this challenging, host-DNA-rich matrix.

The dominant phyla identified in milk samples —*Firmicutes* (recently renamed as *Bacillota*), *Proteobacteria* (renamed as *Pseudomonadota*), and *Actinobacteria* (renamed as *Actinomycetota*)— were consistent with those reported by other authors in bovine milk (Bonsaglia et al., 2017; Urrutia-Angulo et al., 2024; Vasquez et al., 2022). However, it is worth noting the high proportion of unclassified phyla found (50.4%), which underscores the challenge of characterizing the full diversity. This high proportion is likely attributable to the sequencing technology (short vs. long reads) (van Dijk et al., 2018) and approach (metabarcoding vs. shotgun metagenomics) (Ciuffreda et al., 2021) used, as well as to the stringency of the bioinformatic pipeline employed. Specifically, SqueezeMeta applies stringent criteria for taxonomic classification (≥80% of the best hit’s bit-score and within 10% identity difference), assigning a contig to a genus only when the evidence is robust (identity threshold ≥60%) (Tamames and Puente-Sánchez, 2019). This conservative approach, while potentially underestimating diversity at finer taxonomic levels, enhances confidence in the taxonomic calls made.

Antimicrobial DCT was not associated with significant changes in microbial taxonomic diversity or community composition and both treatment groups shared a core-genera composition which likely represent stable members of the milk microbiota regardless of the treatment status. These findings agree with previous studies in animals treated with antimicrobials of the same class (β-lactams) (Basbas et al., 2022; Biscarini et al., 2020; Bonsaglia et al., 2017; Filippone Pavesi et al., 2023; Vasquez et al., 2022). However, they contrast with other studies reporting significant taxonomic differences between T and NT animals, even when β-lactams were used (Derakhshani et al., 2018; Dong et al., 2021; Patangia et al., 2023). Although the intramammary antimicrobial used in this study is designed to remain active for an extended period post-dry-off (with a withdrawal time of 36–37 days), an average interval of 260 days elapsed between dry-off antimicrobial treatment and sampling. In this period, it is likely that the milk microbiota had sufficient time to recover, thereby potentially masking any “short-term” antimicrobial effects. The timing of sample collection represents a limitation, as taking serial samples before and shortly after dry-off would have offered a baseline and a more specific view of the microbial shifts associated with DCT.

Interestingly, *Anaplasma* appeared among the most abundant bacterial genera detected in all milk samples. Although this finding is unexpected, previous studies have also reported the presence of *Anaplasma* DNA in milk from apparently healthy animals (Dela Cruz et al., 2019; Guo et al., 2023; Li et al., 2024; Zhang et al., 2016). This could, in part, be explained by the excretion of the pathogen through somatic cells in milk, particularly leukocytes such as neutrophils and other white blood cell lineages, which are known targets of *Anaplasma* spp (Dela Cruz et al., 2019; WOAH, 2024). In our study, no clinical signs of active infection were recorded in the animals, suggesting that they may be subclinical or persistent carriers of *Anaplasma*, and more specifically of *A. phagocytophilum*, the most frequently detected species in our samples. However, the potential influence of external contamination during sampling or processing, as well as bioinformatic artifacts, cannot be excluded.

In contrast to the relatively stable taxonomic composition, resistome analysis showed that cows treated with antimicrobials at dry-off exhibited a greater diversity and abundance of detected GDRs. These findings reinforce the association between antimicrobial exposure and resistome enrichment in treated cows, even though an average of ~260 days had passed since DCT. A significantly higher relative abundance of ARGs has been reported in milk from treated cows —particularly those receiving cefquinome— with a notable enrichment in genes conferring resistance to cephalosporins, aminoglycosides, and penams-type β-lactam antimicrobials (Patangia et al., 2023). Moreover, the use of antimicrobials such as ceftiofur and cefquinome has been shown to significantly increase the proportion of *bla*_TEM_ genes in milk at the time of withdrawal, highlighting how antimicrobial exposure can modulate the resistome over time, particularly during the early days (3 days) post-treatment (Dong et al., 2021). In our study, *bla*_TEM_ was detected exclusively through ONT sequencing, whereas Illumina sequencing identified only *bla*_CTX_, and at low abundance. However, no clear differences in the abundance or diversity of β-lactam resistance genes were observed between treated and untreated cows in our dataset, suggesting that the presence of these genes may not be a persistent consequence of DCT or may fall below detection thresholds at sampling time points.

Certain GDRs were also found in NT cows, indicating that factors beyond direct antimicrobial exposure at drying-off may influence resistome composition. This aligns with previous findings showing that AMR genes can be detected even in healthy animals without prior antimicrobial treatment (Hoque et al., 2020), suggesting that these genes may persist naturally or be acquired through mechanisms such as horizontal gene transfer or co-selection (Vasquez et al., 2022). Here, as no pre-treatment data were available, we cannot exclude the effect of antimicrobial treatments administered before the lactation period monitored. Still, these results reinforce the notion that the milk microbiota may serve as a reservoir for ARGs, posing a risk of dissemination both within the farm and potentially into the food chain.

The low overall abundance of ARGs is somewhat reassuring, yet the presence of metal and biocide resistance genes, especially in certain high-carrier cows like T_14, underscores the complexity of co-selection pressures in the dairy environment. Such findings need further investigation into individual animal factors (e.g., treatment history, housing conditions) that might explain outlier profiles.

### 4.2 Limited agreement between ONT and Illumina in ARG detection highlights trade-offs in sequencing strategy and pipeline choice

Direct comparison of Illumina short-read and ONT long-read sequencing for milk resistome profiling on the same set of samples revealed significant discrepancies; ONT detected more ARGs than Illumina but overall agreement was low. This discrepancy likely reflects inherent strengths and limitations of each technology. Illumina assemblies produced relatively short contigs (N50 = 850 bp). Such small contigs limit the ability to detect full-length ARGs, particularly those larger than the average contig size, under the applied thresholds (≥90% identity and ≥80% coverage). In contrast, ONT sequencing generated longer reads (N50 = 6,500 bp), increasing the chance of recovering complete ARG sequences, which may enhance detection sensitivity in alignment-based pipelines such as ABRicate. However, longer ONT reads did not translate into reliable detection of SNPs; Illumina’s higher per-base accuracy remains essential for confidently identifying AMR-associated SNPs (Yorki et al., 2023). This was evident in our results, where SNP confirmation was feasible only for Illumina data analyzed with the AMR++ pipeline. A further theoretical advantage of long reads is their ability to resolve the genomic neighborhood of ARGs and link them to their microbial hosts. In our study, host attribution was not reached for any of the Illumina contigs but was also limited for ONT reads, probably due to insufficient sequencing depth. Thus, only three ONT reads met the criteria for confident host assignment, highlighting the challenge of linking ARGs to taxa in low-microbial-load samples. Although outside the scope of this study, several strategies have been shown to improve microbial host assignment of ARGs, such as hybrid assembly and a genomic-centric analysis (Gounot et al., 2022) or the use of ONT-determined DNA methylation patterns to associate them to a potential host (Bloemen et al., 2025). This illustrates the inherent trade-offs between sequencing platforms and emphasizes that no single sequencing approach is universally optimal for resistome characterization (Petrillo et al., 2022).

Beyond the sequencing technologies themselves, our findings also highlight the importance of the bioinformatic approach in the ARGs detection sensitivity. Tools based solely on sequence alignment against reference databases, such as ABRicate, may fail to detect ARGs, particularly in fragmented short-read assemblies, and do not account for the presence of resistance-conferring SNPs (Seemann, 2016). In contrast, pipelines such as AMR++ combine raw read mapping and SNPs verification, and quantify ARG abundances, which is useful for comparative resistome studies (Bonin et al., 2023). However, AMR++ does not support host attribution of ARGs, an advantage that longer ONT reads could theoretically provide. These differences illustrate that the choice of bioinformatic pipeline can strongly shape resistome results and that pairing an appropriate tool with each sequencing technology is crucial to balance detection sensitivity, accuracy, and interpretability.

Despite these technical constraints, ARGs were exclusively (ONT) or mostly (Illumina) detected in samples from antimicrobial-treated cows, supporting the hypothesis that antimicrobial DCT exerts a measurable impact on the milk resistome. While overall ARG abundance remained low (consistent with previous studies (Vasquez et al., 2022)), our results highlight the ongoing analytical challenge of profiling the resistome in a complex, host-DNA-rich and low-biomass matrices like milk and emphasize the need for continued optimization of sequencing and bioinformatic approaches. The low microbial biomass in milk, coupled with the presence of host (bovine) cells and non-cellular components such as proteins and fats, makes it challenging for robust resistome studies. One of the methodological challenges encountered for both short-read and long-read sequencing technologies was the high proportion of host DNA sequenced, which is a common limitation in studies involving low-biomass matrices (Duarte et al., 2024; Ganda et al., 2021; Yap et al., 2020). Various strategies have been proposed to mitigate this issue. Host DNA can be depleted before sequencing using enzymatic treatments or commercial kits (Bloomfield et al., 2023; Duarte et al., 2024). Additionally, enrichment-based strategies such as ARG-targeted capture or ONT adaptative sampling to selectively sequence ARGs can be used (Duarte et al., 2024; Ganda et al., 2021; Rubiola et al., 2020; Warder et al., 2021). After sequencing, bioinformatic tools such as Trimmomatic or Bowtie2 can align and remove host-derived reads (Bolger et al., 2014; Langmead and Salzberg, 2012).

Despite these strategies, the remaining microbial fraction in milk samples typically accounts for only a small percentage of total reads (often less than 10% in healthy or culture-negative samples, and up to around 30% in culture-positive mastitic milk), whereas host DNA still constitutes the majority of the Illumina and ONT sequencing data (Ahmadi et al., 2023; Collis et al., 2024; Santamarina-García et al., 2024). In our study, we did not perform any host depletion before sequencing, as preliminary tests using commercial depletion kits (MolYsisBasic5, Molzym and NEBNext Microbiome DNA enrichment, New England Biolabs) resulted in total DNA concentrations far below the input requirements for sequencing (data not shown). In fact, given that a certain degree of non-specific depletion of prokaryotic DNA cannot be avoided, these methods are not recommended for samples with a low microbe-to-host DNA ratio, such as milk. Moreover, depletion kits can have different specificity for different bacterial taxa (Bhute et al., 2025; Kim et al., 2024). We acknowledge that this decision likely impacted the sequencing depth of the bacterial target and therefore the sensitivity of both taxonomic and resistome profiling. Instead, host DNA was removed bioinformatically after sequencing. Consistent with previous reports, 88.1% of the ONT sequences obtained per sample aligned to the bovine genome after filtering with Bowtie2, leaving only 11.9% of the reads for microbiome and resistome analyses. This excess of bovine DNA significantly reduces the sequencing depth obtained for microbial genomes, ultimately affecting the accuracy of resistome and microbiome characterization (Gweon et al., 2019; Pereira-Marques et al., 2019; Zaheer et al., 2018). Rarefaction analysis of ONT data further confirmed this limitation, as sequencing curves did not reach *plateau*, confirming that sequencing depth was insufficient to fully capture the underlying microbial diversity. Nevertheless, ONT still enabled the detection of a broader variety of ARGs than Illumina, along with a higher proportion of taxonomically classified reads.

Another important consideration is the normalization approach used in our resistome analysis. To facilitate between-sample comparisons, ARG abundances were normalized using CPM. This method allowed comparison of resistome profiles between T and NT cows but cannot be used for absolute quantification to report accurate resistance levels. Alternative pipelines such as ARGs-OAP, which normalizes ARG abundances based on microbial load (e.g.,16S rRNA gene copy number or estimated cell counts), may offer more realistic or biologically meaningful values and comparability across studies (Yin et al., 2023b, 2023a). Lastly, the relatively small sample size further limited the robustness of the findings, warranting caution when interpreting taxonomic profiles and resistome richness based on these data. The insights generated from this exploratory study may provide a valuable foundation for future research in this field. Studies with a larger sample size, pre-treatment baseline data, deeper microbial sequencing, and optimized methodological approaches will be needed to confirm and expand our findings.

## 5 Conclusion

This study adds new insights into the impact of antibiotic DCT on the milk microbiome and resistome. While we found no detectable differences in the overall taxonomic composition of the milk microbiota, antimicrobial DCT was associated with increased prevalence, diversity, and abundance of GDRs in treated cows. While our study was not intended as a formal methodological benchmarking analysis, our integration of Illumina short-read and ONT long-read results underscore that both sequencing technology and bioinformatic pipeline choices critically affect resistome profiling. The high presence of host DNA and low microbial biomass are common challenges in milk metagenomics, independent of the sequencing platform. Although rarefaction curves indicated that our ONT sequencing depth was insufficient to fully capture microbial diversity, the longer reads of the ONT platform provided greater sensitivity for detecting a wider variety of GDRs, an advantage that compensates its lower sequencing depth. This emphasizes the need for optimized host DNA depletion and potential targeted enrichment strategies to improve sequencing performance across both short- and long-read platforms. Taken together, our findings reinforce the importance of combining robust sequencing approaches with adapted analytical workflows to advance accurate resistome surveillance in dairy production. As AMR poses a significant global challenge, these findings advocate the need for cautious antimicrobial use and continued exploration of alternative strategies for mastitis control.

## Data Availability

The datasets presented in this study can be found in online repositories. The names of the repository/repositories and accession number(s) can be found below: https://www.ebi.ac.uk/ena. All raw metagenomic sequencing data generated in this study have been deposited in the European Nucleotide Archive (ENA) under the Study Accession number PRJEB93971. Sample Accession numbers range from ERS25262654 to ERS25262684. Illumina short-read data are available under Run Accession numbers ERR15299398 to ERR15299428, and ONT long-read data under ERR15299429 to ERR15299438 (**Supplementary Table S1**).
