# Supplementary material for: Resistome and microbiome profiling of bovine milk following antimicrobial dry cow therapy: insights from short- and long-read metagenomic sequencing
Source: Front Microbiomes. 2025 Oct 10;4:1672438. doi: 10.3389/frmbi.2025.1672438 (PMC12993662; doi:10.3389/frmbi.2025.1672438)

Supplementary Figures

Resistome and microbiome profiling of bovine milk following antimicrobial dry cow therapy: Insights from short- and long-read metagenomic sequencing

Leire Urrutia-Angulo[1], José Luis Lavín[2], Beatriz Oporto[1], Gorka Aduriz[1], Ana Hurtado[1], Medelin Ocejo[1]*

[1]Animal Health Department, [2]Applied Mathematics Department, NEIKER- Basque Institute for Agricultural Research and Development, Basque Research and Technology Alliance (BRTA), Bizkaia Science and Technology Park 812L, 48160 Derio, Bizkaia, Spain

Supplementary Figure S1. Rarefaction curves plotting the number of genera at different sequencing depths for (A) the 31 milk samples sequenced using Illumina technology and (B) the 10 samples sequenced with ONT after eukaryotic DNA removal. The x-axis represents the sequencing depth in number of reads, and the y-axis shows the number of genera detected.

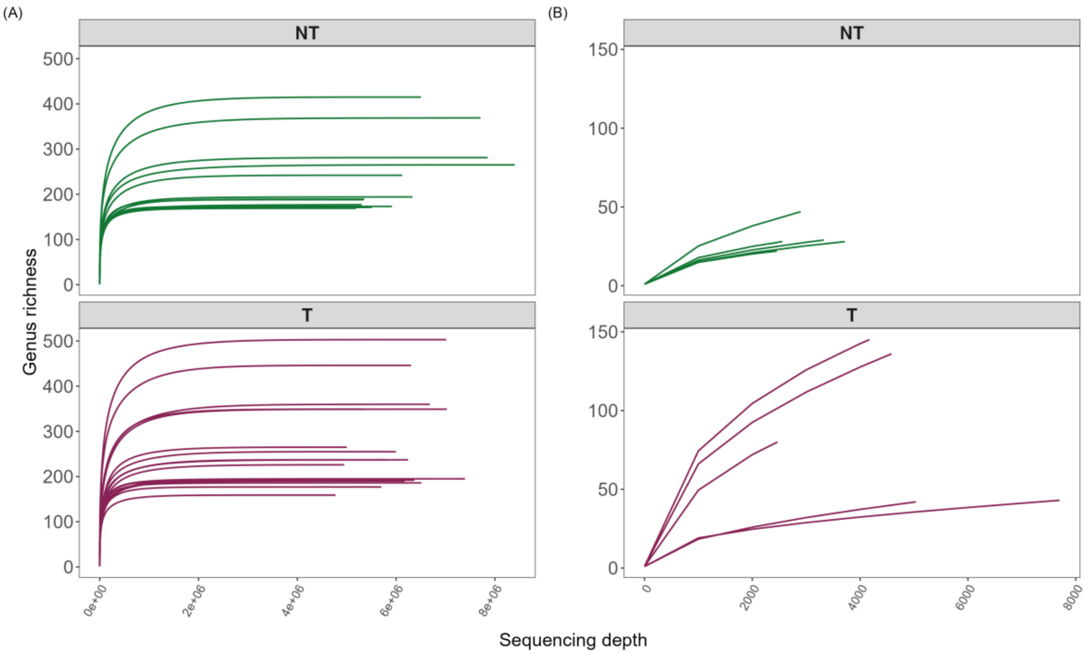

Supplementary Figure S2. Venn diagram showing unique and shared GDRs in milk samples from NT and T animals. The diagram includes both ARGs and SNPs, the latter indicated by an asterisk (*).

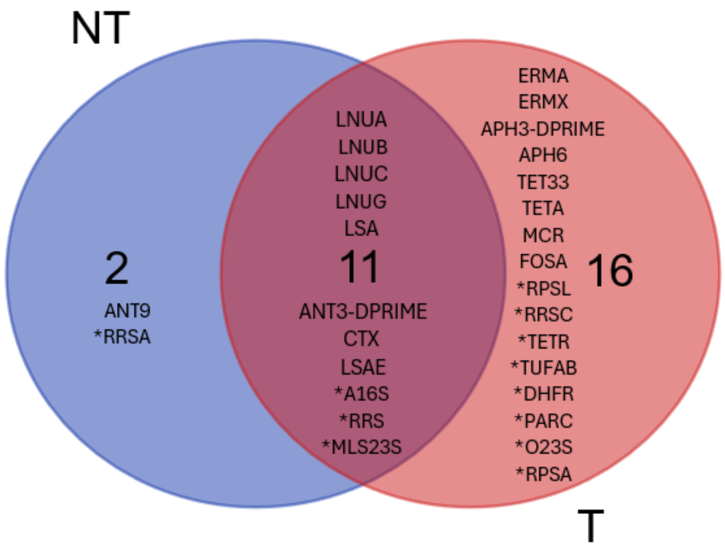

Supplement: Supplementary file 1 [file DataSheet1.pdf]
